# Differential Regulation of PI(4,5)P_2_ Sensitivity of Kv7.2 and Kv7.3 Channels by Calmodulin

**DOI:** 10.3389/fnmol.2017.00117

**Published:** 2017-05-01

**Authors:** Carolina Gomis-Perez, Maria V. Soldovieri, Covadonga Malo, Paolo Ambrosino, Maurizio Taglialatela, Pilar Areso, Alvaro Villarroel

**Affiliations:** ^1^Biofisika Institutua, Consejo Superior de Investigaciones Científicas, CSIC, UPV/EHULeioa, Spain; ^2^Department of Medicine and Health Science, University of MoliseCampobasso, Italy; ^3^Department of Neuroscience, University of Naples “Federico II,”Naples, Italy; ^4^Department Farmacología, UPV/EHU, Universidad del País VascoLeioa, Spain

**Keywords:** apo-calmodulin, PIP_2_, KCNQ, Kv7, M-current

## Abstract

**HIGHLIGHTS**

- Calmodulin-dependent Kv7.2 current density without the need of binding calcium.
- Kv7.2 current density increase is accompanied with resistance to PI(4,5)P_2_ depletion.
- Kv7.3 current density is insensitive to calmodulin elevation.
- Kv7.3 is more sensitive to PI(4,5)P_2_ depletion in the presence of calmodulin.
- Apo-calmodulin influences PI(4,5)P_2_ dependence in a subunit specific manner.

The identification and understanding of critical factors regulating M-current functional density, whose main components are Kv7.2 and Kv7.3 subunits, has profound pathophysiological impact given the important role of the M-current in neuronal excitability control. We report the increase in current density of Kv7.2 channels by calmodulin (CaM) and by a mutant CaM unable to bind Ca^2+^ (CaM_1234_) revealing that this potentiation is calcium independent. Furthermore, after co-expressing a CaM binding protein (CaM sponge) to reduce CaM cellular availability, Kv7.2 current density was reduced. Current inhibition after transient depletion of the essential Kv7 co-factor phosphatidylinositol-4,5-bisphosphate (PI(4,5)P_2_) by activating *Danio rerio* voltage sensitive phosphatase (DrVSP) was blunted by co-expressing CaM_1234_ or the CaM sponge. In addition, CaM-dependent potentiation was occluded by tonic elevation of PI(4,5)P_2_ levels by PI(4)P5-kinase (PIP5K) expression. In contrast to the effect on homomeric Kv7.2 channels, CaM_1234_ failed to potentiate heteromeric Kv7.2/3 or homomeric Kv7.3 channels. Sensitivity to PI(4,5)P_2_ depletion of Kv7.2/3 channels was increased after expression of CaM_1234_ or the CaM sponge, while that of homomeric Kv7.3 was unaltered. Altogether, the data reveal that apo-CaM influences PI(4,5)P_2_ dependence of Kv7.2, Kv7.2/3, and of Kv7.3 channels in a subunit specific manner.

## Introduction

Calmodulin (CaM) is a small acidic protein of 148 residues that confers Ca^2+^ sensitivity to a large variety of intracellular proteins, although Ca^2+^ independent roles for CaM are also emerging (Zhang et al., 1995; Jurado et al., 1999; Villarroel et al., 2014). The importance of CaM in the central nervous system (CNS) function is reflected in the extraordinary high concentration of this protein (from 10 to 100 μM) in different brain areas (Xia and Storm, 2005). However, several functional studies suggest that free apoCaM concentration is in the submicromolar range (Sanabria et al., 2008; Zhong et al., 2009; Alaimo et al., 2013; Bossuyt and Bers, 2013), because CaM availability (free CaM) depends on the concentration and phosphorylation state of several CaM-binding proteins (Alexander et al., 1988; Xia and Storm, 2005) thus little is known about the consequences of free CaM oscillations.

Kv7.2 and Kv7.3 subunits, either forming Kv7.2 homotetramers, or Kv7.2/3 heterotetramers are the main component of the neuronal M-current, a non-inactivating voltage dependent potassium conductance that operates in the subthreshold voltage range of action potential generation. CaM mediates Ca^2+^-dependent M-current regulation by bradykinin activation in sympathetic neurons (Gamper and Shapiro, 2003; Greene and Hoshi, 2017) and influences the action of the essential co-factor phosphatidylinositol 4,5-bisphosphate (PI(4,5)P_2_) (Kosenko et al., 2012; Kang et al., 2014; Alberdi et al., 2015). In addition, the reduction in free CaM in hippocampal neurons decreases M-current density and increases neuronal excitability (Shahidullah et al., 2005; Zhou et al., 2016), whereas elevation of CaM levels in sympathetic neurons prevents bradykinin M-current suppression (Zaika et al., 2007).

Here, we have examined whether changes in CaM availability modify the functionality and PI(4,5)P_2_ regulation of Kv7.2 and Kv7.3 subunits. We found that expression of a CaM variant unable to bind Ca^2+^ (CaM_1234_) selectively makes Kv7.2 channels more resistant to PI(4,5)P_2_ depletion, whereas Kv7.2/3 channels become more susceptible to PI(4,5)P_2_ reduction. Moreover, although the sensitivity of homomeric Kv7.3 channels to alterations of PI(4,5)P_2_ is unaffected, CaM still caused a leftward shift on their conductance-voltage relationship. Thus, the present results suggest the existence of an inverted bell-shaped relationship between apoCaM availability and PI(4,5)P_2_ sensitivity in Kv7.2, such that there is an increased resistance to PI(4,5)P_2_ depletion both under low and high apoCaM conditions. In contrast, Kv7.2/3 channels present a mirror behavior, such that the dependency on PI(4,5)P_2_ decreases under low and high apoCaM conditions. Since many channels that bind apoCaM are also regulated by PI(4,5)P_2_ (Suh and Hille, 2008; Villarroel et al., 2014) the influence of apoCaM on PI(4,5)P_2_ affinity likely represents a form of ion channel regulation whose implication goes beyond the Kv7 family.

## Methods

### Molecular biology

The human isoform 3 Kv7.2 (Y15065) and Kv7.3 (NM004519) cDNAs were provided by T. Jentsch (Leibniz-Institut für Molekulare Pharmakologie, Berlin, Germany) and the cDNA encoding rat CaM was provided by the group of J.P. Adelman (Vollum Institute, Portland, OR, USA). The subunits tagged at the N-terminal with mCFP or mYFP were cloned into pCDNA3.1 and we previously confirmed that these N-terminal tags have no impact on the electrophysiological properties of the channel (Gómez-Posada et al., 2011). Point mutations were constructed by PCR-based mutagenesis. The Kv7.3 A315T construct has been described previously (Gómez-Posada et al., 2010). The CaM sponge, that has CFP and YFP flanking the apoCaM-binding site of neuromodulin, was provided by D.J. Black (University of Missouri, Missouri, USA), PI(4)P5-kinase type IV was provided by B. Hille (University of Washington, Seattle, USA) and Dr-VSP-IRES-GFP from zebrafish was provided by Y. Okamura (Osaka University, Osaka, Japan).

### Cell culture and transfection

HEK293T cells (HEK 293T/17, ATCC, CRL-11268) were maintained in 5% CO_2_ at 37°C in Dulbecco's Modified Eagle's Medium (DMEM, Sigma-Aldrich), supplemented with non-essential amino acids (Sigma, Madrid, Spain) and 10% FBS (Lonza, Madrid, Spain).

### Electrophysiological measurements

Whole-cell patch-clamp recordings of HEK293T cells were obtained at RT (21–25°C) 48 h after transfection using lipofectamine 2000 (Invitrogen). Cells were bathed in the following solution (mM): 140 NaCl, 4 KCl, 2 CaCl_2_, 2 MgCl_2_, 10 HEPES (Na), 5 D-glucose, adjusted to pH 7.4 with NaOH. The osmolarity was adjusted with mannitol to ~315 mOsm. Pipettes were pulled from borosilicate glass capillaries (Sutter Instruments, USA) using a Narishige micro-pipette puller (PC-10; Narishige Instrument Co., Japan). Membrane currents were measured using an EPC-8 amplifier (HEKA Instruments, Germany) with >80% series resistance compensation, pipette and membrane capacitance cancellation. Cells with similar fluorescent intensity were selected. All experiments to test the impact of DrVSP (0.5 μg cDNA per 35 mm dish) were carried out with 100% series resistance compensation using a VE-2 amplifier (Alembic Instruments, Canada) equipped with Rs Compensator (Sherman et al., 1999).

Pipettes were filled with an internal solution containing (mM): 125 KCl, 10 HEPES (K), 5 MgCl_2_, 5 EGTA, 5 Na_2_ATP, adjusted to pH 7.2 with KOH and the osmolarity adjusted to ~300 mOsm with mannitol. The amplitude of the Kv7 current was defined as the peak difference in current relaxation measured at −30 mV after 500–1,500 ms pulses to −110 mV (all channels closed) and to +30 mV (all channels opened). This protocol subtracts all leak currents from the measurements. The data were acquired and analyzed using pCLAMP software (version 8.2), normalized in Excel (Microsoft Corp., Madrid, Spain) and plotted using SigmaPlot (SPSS Corp., Madrid, Spain).

### Statistics

Data are shown as the mean ± S.E.M. The differences between the means were evaluated using the unpaired Student's *t*-test, where values of *P* ≤ 0.05 were considered significant. The number of cells in each experiment is indicated in brackets in the figures. The results are from two or more independent batches of cells. In all figures ^*^, ^**^, and ^***^ indicate significance at the *p* < 0.05, *p* < 0.01, and *p* < 0.001, respectively.

## Results

### Calmodulin potentiates Kv7.2 currents

**Figure 1:**
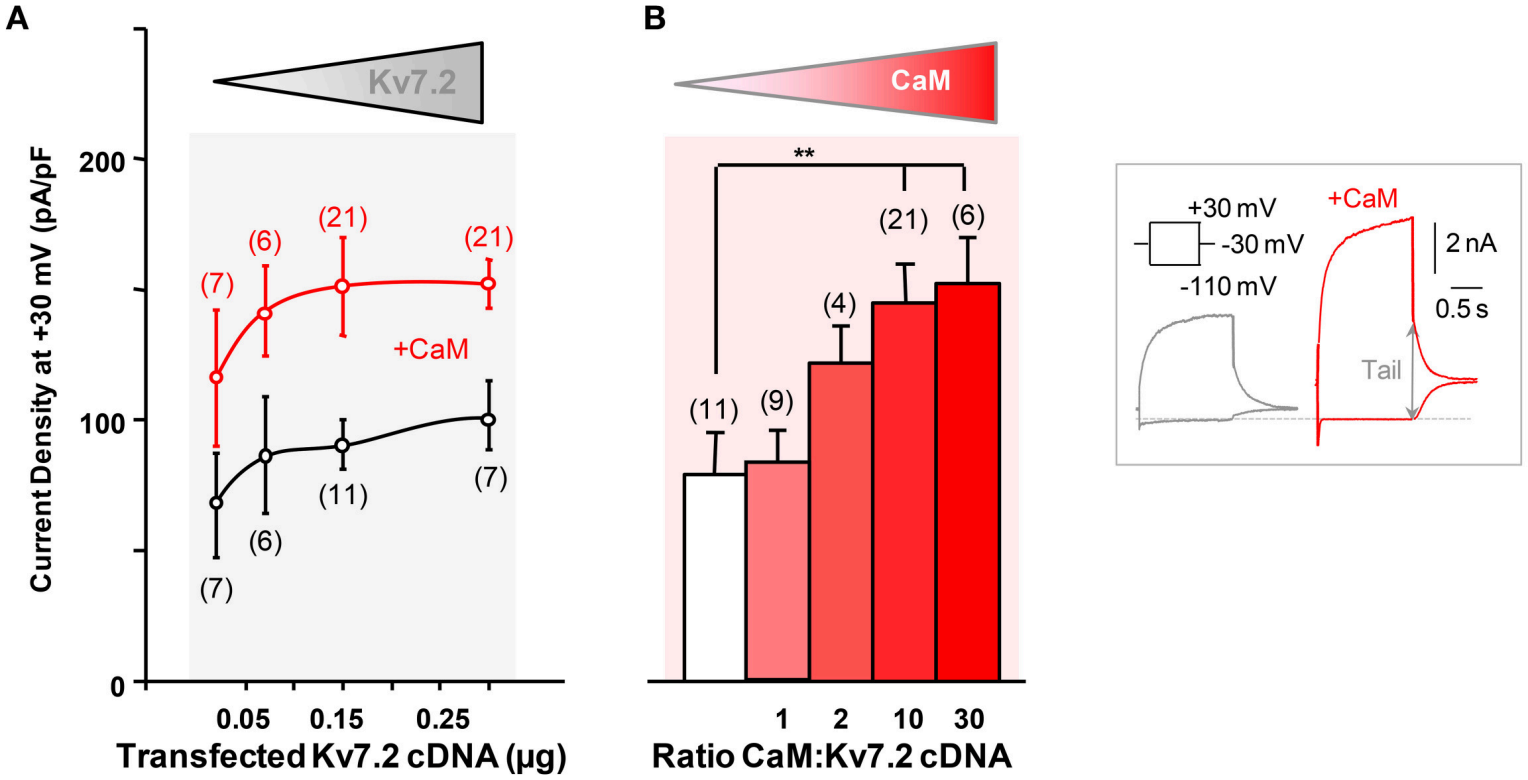
**Characterization of calmodulin-dependent Kv7.2 potentiation. (A)** Effect of CaM on Kv7.2 current density as a function of the amount of channel cDNA transfected. Mean current density (pA/pF) in cells transfected with channels only (black symbols) or together with 3 μg CaM cDNA per 35 mm dish (red symbols). **(B)** Effect of increasing CaM on Kv7.2 density. Half maximal CaM effect was obtained approximately when transfecting cells at a 1:2 (w/w) Kv7.2/CaM cDNA ratio. Inset: Representative current traces recorded from HEK293T cells transfected with 0.15 μg Kv7.2 cDNA and, where indicated, with 3 μg CaM cDNA, to illustrate current density quantification. Maximum current was measured at −30 mV as the difference in the amplitude after a pre-pulse to −110 mV to close all channels and after a prepulse to +30 mV to reach maximum P_open_ (arrow).

The impact of CaM elevation on the function of Kv7 channels has been studied before, often with contrasting results (Gamper et al., 2005; Xu et al., 2007; Zaika et al., 2007; Alaimo et al., 2013; Kang et al., 2014). In the present experiments, we observed that CaM co-expression potentiated the maximal current density of Kv7.2 isoform 3 channels expressed in HEK293T cells (Figure 1) and in CHO cells (data not shown) (Ambrosino et al., 2015); similar results were also achieved when the human isoform 4 of Kv7.2 was expressed (data not shown). In these experiments, to monitor the expression of the channel, isoform 3 was tagged at the N-terminus with CFP; this manipulation has been previously shown not to influence channel function, as the current size and the gating properties of tagged subunits were undistinguishable from those of untagged subunits (Soldovieri et al., 2006). We evaluated next the electrophysiological consequences of transfecting increasing amounts of Kv7.2 cDNA in HEK293T cells by whole-cell patch-clamp. The experiments revealed that the Kv7.2 current density was relatively constant irrespective of the amount of Kv7.2 cDNA transfected (Figure 1A), reaching a maximal value of about 75 pA/pF. The density roughly doubled when CaM was co-expressed, attaining a maximum of approximately 150 pA/pF (Figure 1A). The response to increasing amounts of CaM revealed that half of the maximal density was obtained at a 1:2 Kv7.2/CaM cDNA ratio, whereas no significant effect was detected at a 1:1 ratio (Figure 1B; Alaimo et al., 2013).

**Figure 2:**
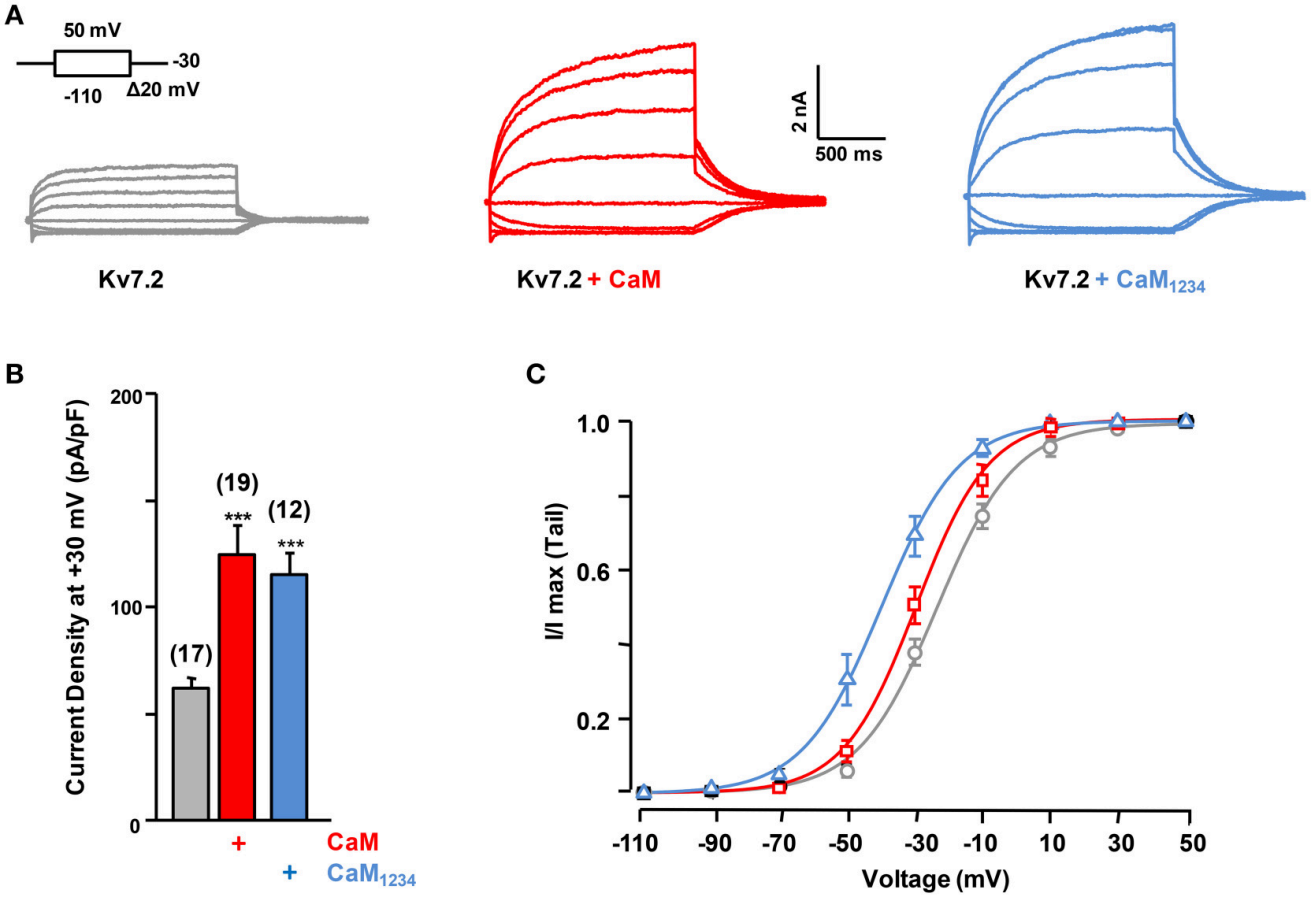
**Effect of calmodulin on Kv7.2 current density. (A)** Representative current traces measured in response to the indicated voltage protocol in cells expressing Kv7.2, Kv7.2 + CaM or Kv7.2 + CaM_1234_. **(B)** Comparison of the effect on current density of endogenous CaM (gray) WT CaM (red), CaM_1234_ (blue). **(C)** Voltage-dependence of activation measured under, resting (black) and elevated (red, CaM_WT_ and blue CaM_1234_) CaM levels. The V_1/2_ (mV) under resting and elevated CaM_WT_ and CaM_1234_ conditions were (number of experiments in brackets): −23.2 ± 0.8 (10), −29.4 ± 1.1 (8), and −39.4 ± 1.2 (8).

To address the requirement of Ca^2+^ binding to CaM, the effect of CaM_1234_ was assessed. CaM_1234_ harbors D>A substitutions at each of the four EF-hands, preventing Ca^2+^ binding (Putkey et al., 1989). The extents of current potentiation observed with CaM and CaM_1234_ were indistinguishable (Figures 2A,B), although CaM_1234_ overexpression caused a leftward shift in the voltage dependence of activation of Kv7.2 channels (Figure 2C). Thus, the increase in current density does not require Ca^2+^ binding to CaM (Ambrosino et al., 2015).

**Figure 3:**
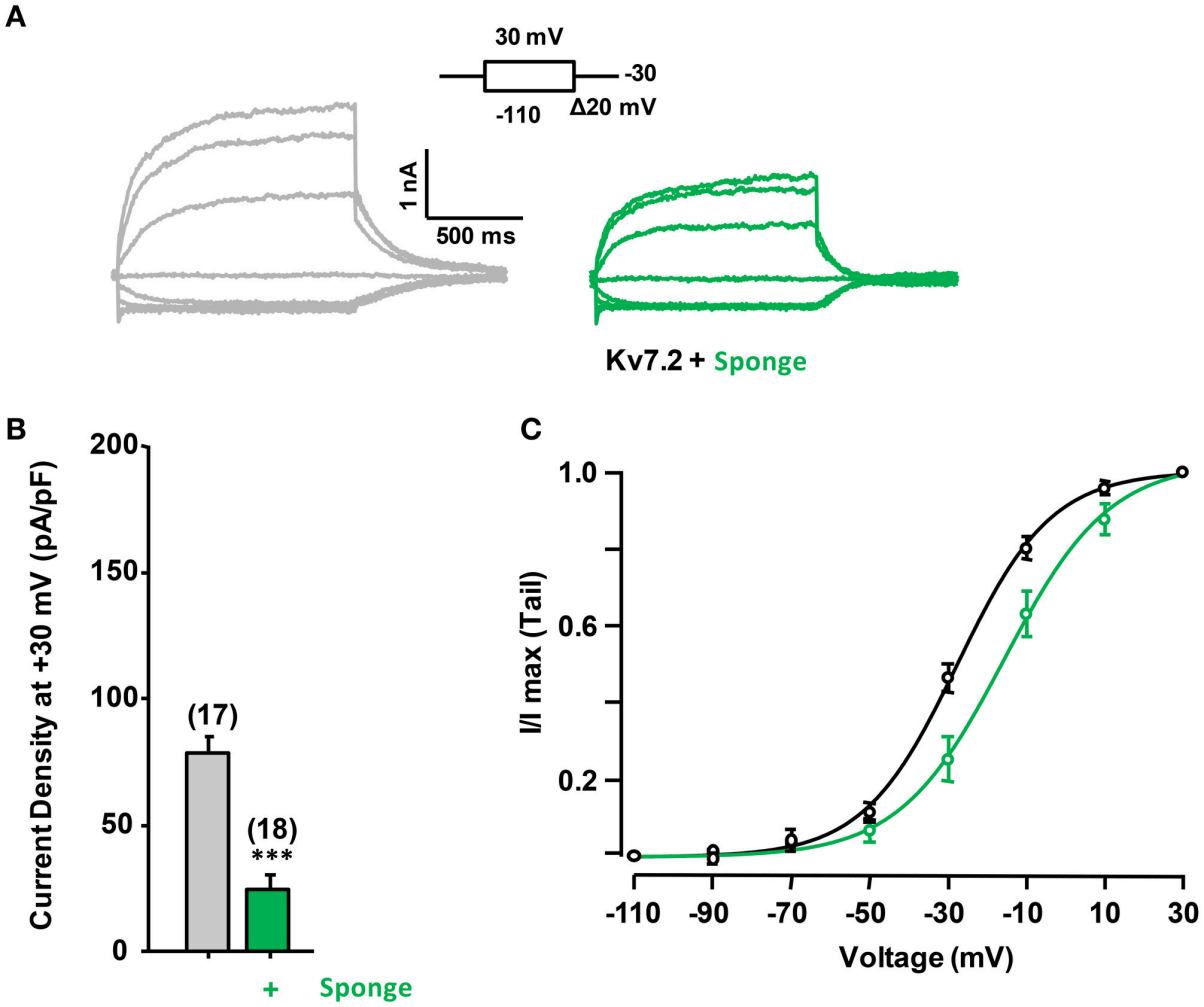
**Effect of reduced calmodulin levels on Kv7.2 current density. (A)** Representative current traces measured in response to the indicated voltage protocol in cells expressing Kv7.2 or Kv7.2 + neurogranin (sponge, green). **(B)** Comparison of the effect on current density of endogenous CaM (gray) and reduced CaM levels (+Sponge, Green). **(C)** The V_1/2_ (mV) under reduced and resting CaM conditions were (number of experiments in brackets): −27.8 ± 0.9 (8) and −15.6 ± 2.1 (8).

To address the consequences of reduced CaM levels we over-expressed a CaM binding protein (CaM sponge, Figure 3; Black et al., 2004; Liu et al., 2010). This intervention led to a decrease in Kv7.2 current density (Figure 3) and to a right shift in the voltage-dependence (Figure 3C). When both the CaM sponge and CaM_1234_ were co-expressed the current density was similar to control (data not shown), supporting the notion that the effect of the CaM-sponge (neurogranin) on Kv7.2 was due to reduced CaM availability.

**Figure 4:**
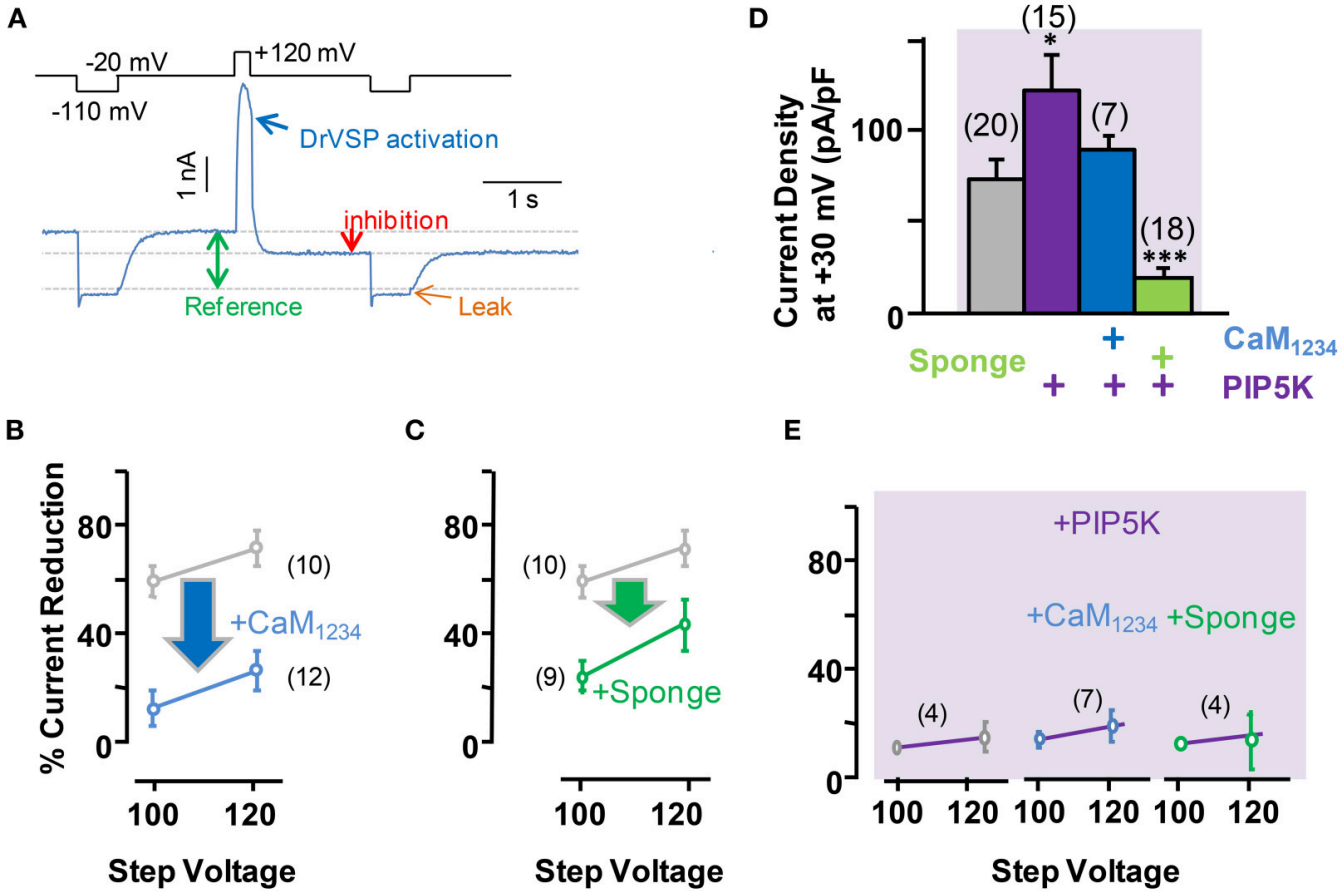
**Effect of acute reduction of PI(4,5)P_**2**_ levels on calmodulin-dependent regulation of Kv7.2 current density. (A)** Scheme of the voltage protocol used to illustrate current inhibition quantification. **(B)** Averaged current reduction after activation of DrVSP at +100 or +120 mV under resting conditions (gray symbols) and with CaM_1234_ (blue symbols). **(C)** Averaged current reduction in the presence of the CaM sponge (green symbols). **(D)** Comparison of current density with CaM_1234_ (blue) and the CaM-sponge (green) in cells co-expressing PIP5K (purple). **(E)** Averaged current reduction after activation of DrVSP in the presence PIP5K in control and cells co-expressing CaM_1234_ or the CaM-sponge (magenta lines).

To evaluate the role of PI(4,5)P_2_ in Ca^2+^-independent CaM regulation of Kv7.2 currents, we used *Danio rerio* voltage-dependent phosphatase (DrVSP), which catalyzes the removal of a phosphate group at position 5, resulting in the rapid depletion of PI(4,5)P_2_. DrVSP is barely active at the voltages used to quantify Kv7 voltage-evoked current relaxations (Falkenburger et al., 2010), but becomes activated at highly-depolarized voltages (Figure 4A). The consequence of elevation (CaM_1234_ co-expression) or reduction (CaM sponge co-expression) of CaM levels on DrVSP sensitivity of Kv7.2 currents was investigated (Figure 4). The results obtained revealed that activation of DrVSP was less effective at reducing Kv7.2 currents under both experimental conditions (Figures 4B,C, respectively), leading to the surprising conclusion that both increased and reduced CaM levels rendered Kv7.2 channels more resistant to PI(4,5)P_2_ decrease. Similar results were obtained when untagged Kv7.2 subunits were tested (data not shown).

The role of PI(4,5)P_2_ was further evaluated by co-expressing a PI(4)P5-kinase (PIP5K, Figures 4D,E, see also Supplemental Figure 1), which has been shown to increase Kv7.2 current density as a consequence of the tonic elevation of PI(4,5)P_2_ levels (Li et al., 2005; Winks et al., 2005; Soldovieri et al., 2016). This effect has been attribute to an increased channel open probability, which is strongly regulated by PI(4,5)P_2_ (Li et al., 2005). Consistent with these previous reports, co-expression of PIP5K in HEK293 cells almost doubled Kv7.2 current density (Figure 4D). However, simultaneous expression of PIP5K and CaM resulted in current densities that where smaller than when PIP5K was expressed alone. On the other hand, expression of PIP5K failed to increase Kv7.2 current density when the levels of CaM were reduced by co-expressing the CaM-sponge (Figure 4D). Finally, co-expression of PIP5K resulted in a remarkable resistance to PI(4,5)P_2_ depletion, under resting, elevated and reduced CaM levels (Figure 4E), a result consistent with the concept that expression of PIP5K raised PI(4,5)P_2_ to concentrations beyond those required to saturate Kv7.2 channels.

### The F24A Kv7.2 mutant presents larger current density than wild type channels and is insensitive to calmodulin regulation

**Figure 5:**
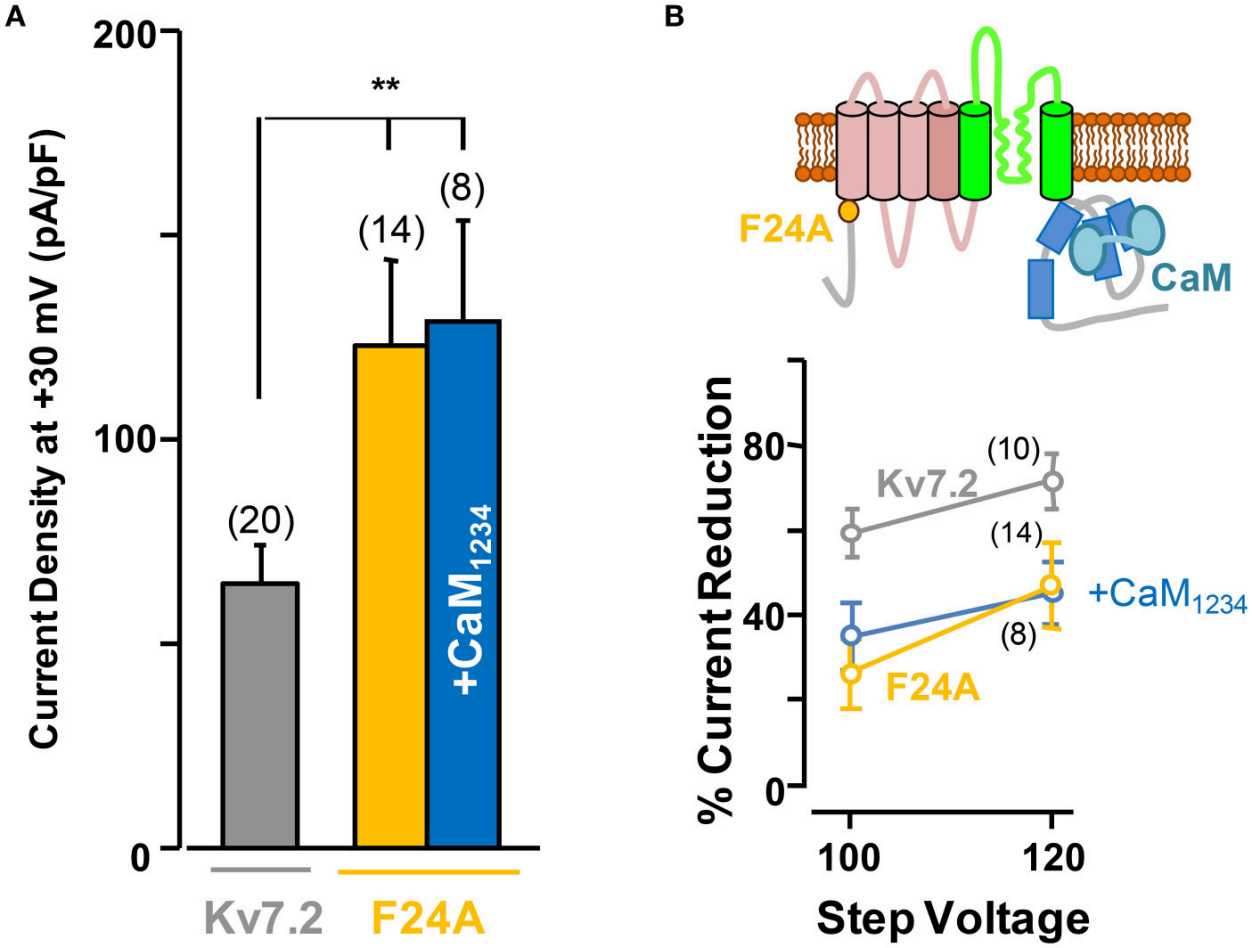
**The F24A mutation results in larger current density and increased resistance to acute PI(4,5)P_**2**_ depletion independently of CaM elevation. (A)** Averaged current density of Kv7.2 channels (gray column), F24A mutation under resting conditions (orange) and co-expressing CaM_1234_ (blue). **(B)** Top, cartoon of a channel subunit. Bottom, sensitivity of the F24A mutant to DrVSP activation under resting conditions (orange), and with co-expression of CaM_1234_ (blue). The reference effect on wt Kv7.2 is plotted in gray color.

Overall, these results show that Ca^2+^-independent CaM-mediated Kv7.2 potentiation depends on PI(4,5)P_2_. This can be due to a general effect of CaM on the plasma membrane levels of PI(4,5)P_2_, or it might depend on a direct interaction of CaM with Kv7.2. CaM binding to Kv7.2 is improved by CaM phosphorylation which makes the channels more resistant to PI(4,5)P_2_ reduction (Kang et al., 2014). CaM phosphorylation is counterbalanced by a resident protein phosphatase 1 (PP1) that binds to the Kv7.2 N-terminus. To unveil if this mechanism is involved in potentiation, we tested the effect of the Kv7.2 N-terminal F24A mutation, which destroys the PP1 binding site (Kang et al., 2014). The results from these experiments revealed that Kv7.2 channels carrying this mutation displayed an increased current density and, importantly, were insensitive to CaM_1234_ elevation (Figure 5A). Furthermore, F24A channels were less sensitive than wt Kv7.2 to the inhibitory effects of DrVSP activation, and this phenomenon persisted under elevated CaM_1234_ conditions (Figure 5B).

### Subunit specificity of calmodulin-dependent potentiation

**Figure 6:**
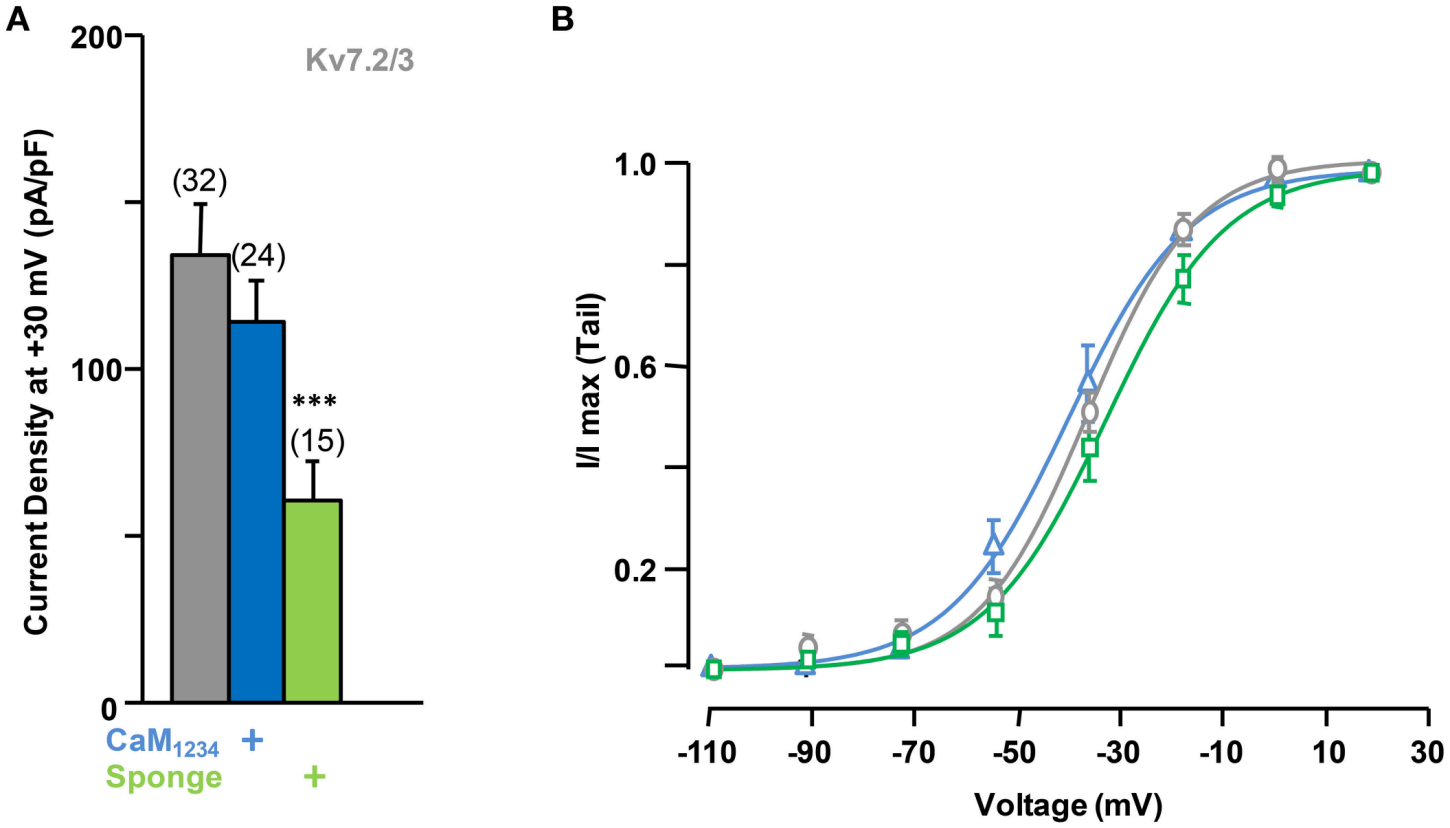
**Effect of alterations of calmodulin levels on Kv7.2/3 current density. (A)** Comparison of the effect on current density of CaM_1234_ (blue) and the CaM-sponge (green). **(B)** Activation voltage-dependency measured under reduced (green), resting (gray) and elevated (blue, CaM_1234_) CaM levels. The V_1/2_ (mV) under low, resting and elevated CaM conditions were (number of experiments in brackets): −26.5 ± 2.0 (7), −30.5 ± 1.2 (9), and −34.1 ± 1.7 (6).

In many neuronal compartments Kv7.2 heteromerize with Kv7.3 subunits, prompting us to examine whether the observed CaM-induced regulation also occurred in Kv7.2/3 heteromers. In contrast to the effect observed for Kv7.2, CaM (data not show) or CaM_1234_ co-expression failed to increase the currents carried by Kv7.2/3 channels (Figure 6A).

**Figure 7:**
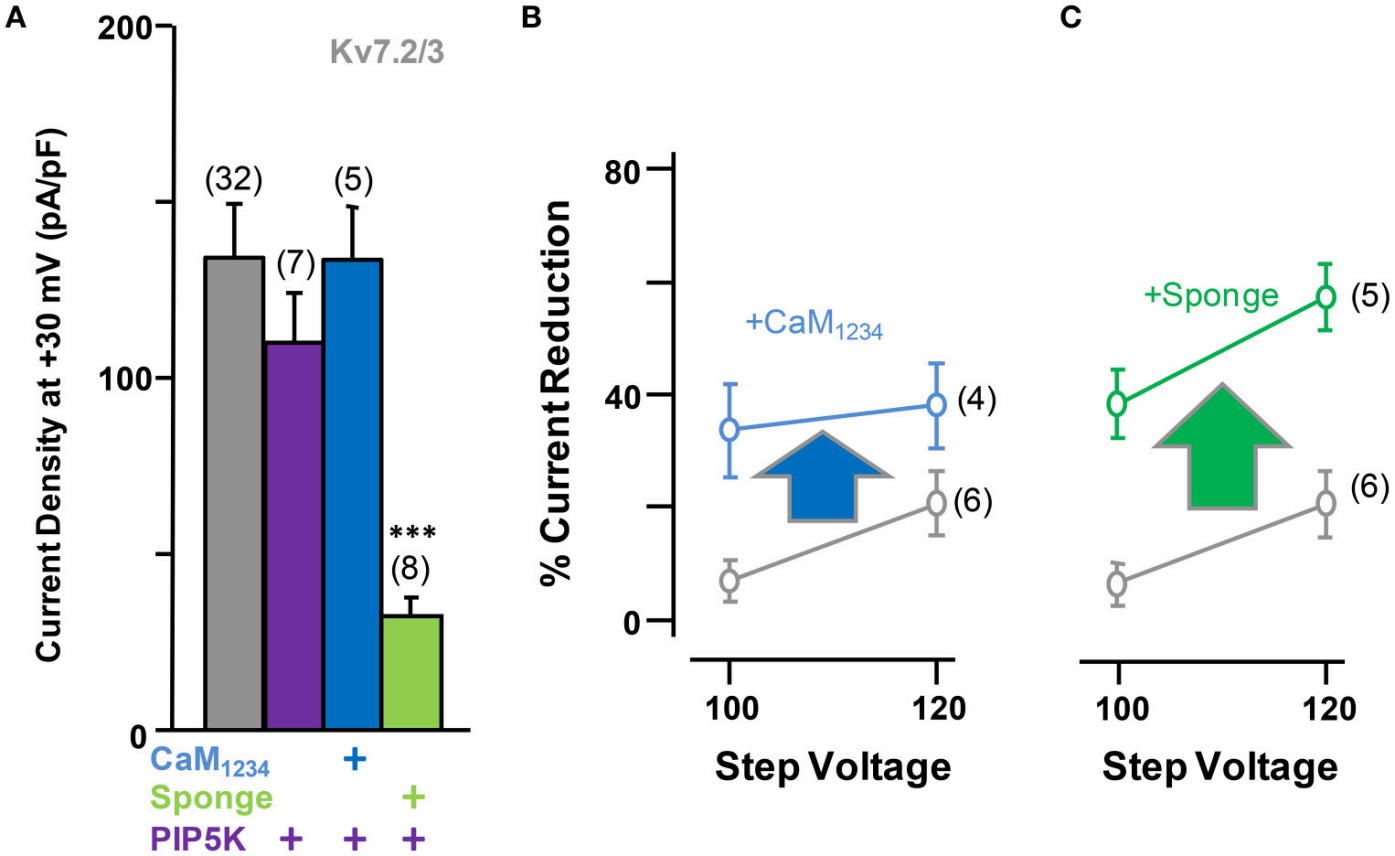
**Effect of alterations of PI(4,5)P_**2**_ levels on calmodulin-dependent regulation of Kv7.2/3 current density. (A)** Comparison of the effect on current density of CaM_1234_ (blue) and the CaM-sponge (green) in cells co-expressing PIP5K (purple). **(B)** Averaged current reduction after activation of DrVSP at different voltages in resting conditions (gray symbols) and with CaM_1234_ (blue symbols). **(C)** Averaged current reduction after activation of DrVSP in resting conditions (gray symbols) and with a CaM sponge (green symbols).

Elevation of tonic PI(4,5)P_2_ levels by PIP5K, alone or in combination with CaM_1234_, did not result in significant changes in current density from Kv7.2/3 channels (Figure 7A). However, similar to the effect observed on Kv7.2 homomeric channels, overexpression of the CaM sponge to reduce tonic CaM levels led to a decrease in Kv7.2/3 current density that was not counterbalanced by PIP5K co-expression (Figures 6A, 7A, see also Supplementary Figure 1). Instead, the density in the presence of the sponge was significantly reduced when PIP5K was co-expressed (Compare Figures 6A, 7A). Notably, the relationship between conductance and voltage was not significantly altered by manipulations of CaM levels (Figure 6B). Moreover, when compared to homomeric Kv7.2 channels, DrVSP activation caused a much smaller decrease in Kv7.2/3 current size, and either CaM_1234_ co-expression or depletion (CaM sponge) increased, rather than decreased as in Kv7.2 channels, the effects of DrVSP activation (Figures 7B,C). Thus, incorporating Kv7.3 subunits in heteromeric channels led to a reversal of the response to tonic CaM levels.

**Figure 8:**
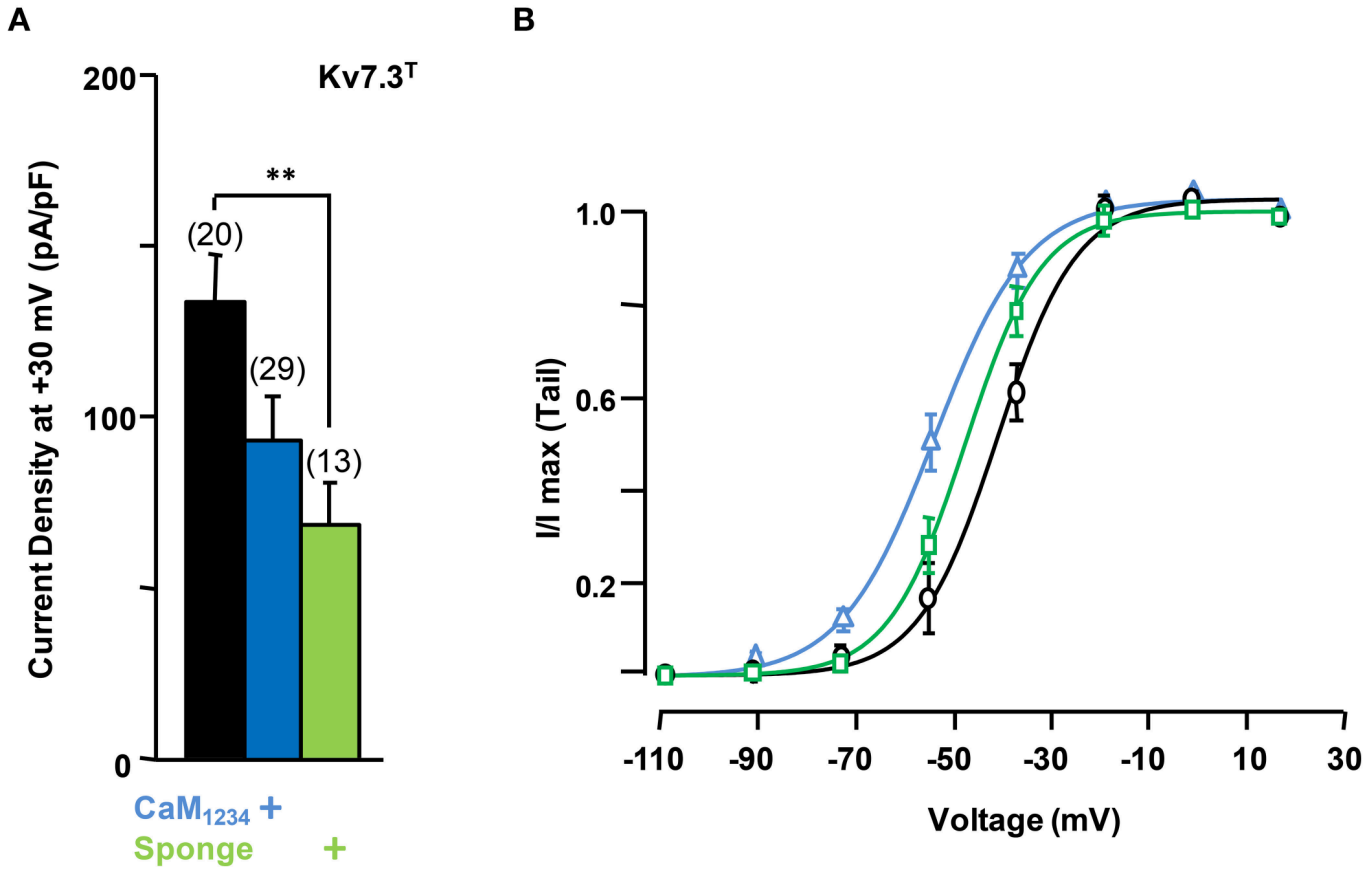
**Effect of calmodulin on Kv7.3^**T**^ current density. (A)** Comparison of the effect on current density CaM_1234_ (blue) and the CaM-sponge (green) on Kv7.3^T^. **(B)** Activation voltage-dependency measured under reduced (green), resting (gray) and elevated (blue, CaM_1234_) CaM levels. The V_1/2_ (mV) under low, resting and elevated CaM conditions were (number of experiments in brackets): −41 ± 1.1 (10), −34 ± 1.4 (11), and −49 ± 1.1 (10).

To gain further insights into this mechanism, we studied homomeric Kv7.3 channels (Figure 8). To this aim, the A315T Kv7.3 variant, denoted Kv7.3^T^, was used because Kv7.3 yields barely detectable currents (Etxeberria et al., 2004; Gómez-Posada et al., 2010). Over-expression of CaM (not shown) or CaM_1234_ failed to augment current density of Kv7.3^T^ channels; rather, this perturbation tended to cause a reduction (Figure 8A).

**Figure 9:**
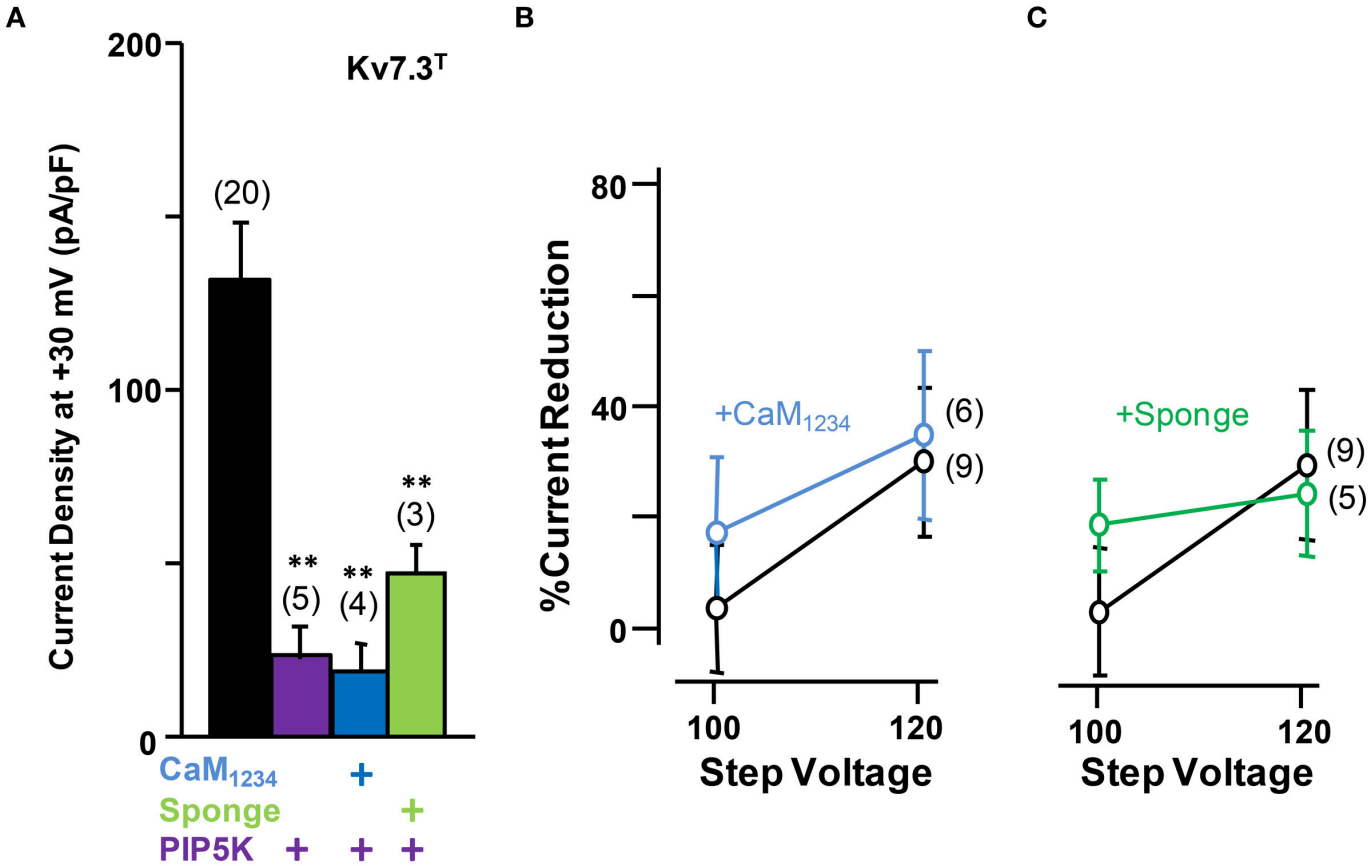
**Effect alterations of PI(4,5)P_**2**_ levels on calmodulin-dependent regulation of Kv7.3^**T**^ current density. (A)** Comparison of the effect on current density of CaM_1234_ (blue) and the CaM-sponge (green) in cells co-expressing PIP5K (purple). **(B)** Averaged current reduction after activation of DrVSP at different voltages in resting conditions (gray symbols) and with CaM_1234_ (blue symbols). (C) Averaged current reduction after activation of DrVSP in resting conditions (gray symbols) and with a CaM sponge (green symbols).

On the other hand, similar to Kv7.2 homomers and Kv7.2/3 heteromers, the density of Kv7.3^T^ was significantly reduced in the presence of the CaM sponge (Figure 8A), and this reduction was not counterbalanced by the expression of PIP5K (Figure 9); furthermore, PIP5K alone also reduced Kv7.3T current density. Noteworthy, a significant leftward shift in the conductance-voltage relationship of Kv7.3^T^ channels was observed upon CaM_1234_ co-expression (Figure 8B). An 84% reduction in Kv7.3^T^ current density was observed as a consequence of PIP5K expression (Figure 9A), accompanied by a leftward shift of the conductance-voltage relationship (Supplementary Figure 1). Similar results on both current density and gating were observed when homomeric channels formed by either Kv7.3 or Kv7.3T subunits were expressed in CHO cells (data not shown). In addition, Western-blot experiments revealed a clear reduction in the amount of Kv7.3 or Kv7.3^T^ subunits in both total lysates and plasma membrane-isolated fraction from CHO cells (data not shown). Finally, no significant changes in the sensitivity to acute PI(4,5)P_2_ depletion (DrVSP activation) was observed after elevation or reduction of CaM levels (Figures 9B,C).

## Discussion

We have investigated in this report the consequences of persistent changes in CaM availability, and the requirement for Ca^2+^ binding using a CaM mutant unable to bind this cation. Based on the similarity of the effects observed with CaM and CaM_1234_ and the changes in current density under conditions that result in tonic elevation or transient reduction of PI(4,5)P_2_ levels, we propose that CaM increases the efficacy of PI(4,5)P_2_ action on Kv7.2 channels without the need of binding Ca^2+^. Remarkably, the potentiation is specific for homomeric Kv7.2 channels. The response to transient depletion of PI(4,5)P_2_ was blunted under conditions that either elevated or reduced CaM availability for Kv7.2, whereas an opposite effect was observed for Kv7.2/3, and no changes occurred in Kv7.3 channels.

Our conclusions agree with and extend those of Kang et al. (2014), who showed that CK2-mediated CaM phosphorylation increased resistance to PI(4,5)P_2_ depletion, although they did not report an increase in current density, probably because the Kv7.2/CaM ratio achieved was lower that investigate in the present report. These authors have shown that protein phosphatase 1 (PP1) is complexed with Kv7.2, and that the F24A mutation disrupts this interaction (Kang et al., 2014). We favor the idea that the larger current density observed for the F24A mutant is due to an increase in PI(4,5)P_2_ efficacy, and, taking into account that occlusion of CaM-dependent potentiation is an indication of mechanistic convergence, we propose that elevation of tonic apoCaM levels increases PI(4,5)P_2_ efficacy for Kv7.2 activity. By the same token, PI(4,5)P_2_ efficacy for Kv7.2/3 is decreased, as revealed by reduced resistance to the action of DrVSP. In contrast, no changes in PI(4,5)P_2_ efficacy were detected for Kv7.3 channels.

One unsolved question is whether the heterogeneous functional consequences observed upon activation of the voltage-dependent phosphatase in the different channels reflect changes in PI(4,5)P_2_ binding affinity. This is an especially challenging question when dealing with ion channels with low apparent PI(4,5)P_2_ binding affinity, such as Kv7.2. Thus, far, the site(s) of interaction is uncertain, since all the mutagenesis data can be explained both as direct or indirect effects on the binding site. Both a change in PI(4,5)P_2_ binding affinity or facilitation of gating transitions downstream PI(4,5)P_2_ binging leading to channel opening can result in CaM-dependent Kv7.2 potentiation and resistance to PI(4,5)P_2_ depletion. Considering that CaM affects multiple cellular processes, such as kinase activity which in turn can also regulate Kv7 channels and its relationship with PI(4,5)P_2_, the data presented cannot exclude an indirect effect. Despite such limitation, the subunit specificity and the impact of the F24A mutation suggest that the consequences described are related to a direct engagement of CaM with the channel, and we favor the idea that this, in turn, affects the interaction of the channel with PI(4,5)P_2_. Solving this issue will require structural information that is presently lacking. The change in PI(4,5)P_2_ efficacy is suggestive that the CaM binding domain can adopt at least two configurations, which confer lower and higher apparent affinity. Considering that the effects of DrVSP on Kv7.2/3 and Kv7.2+CaM are comparable, and taking the reported sensitivity for a PI(4,5)P_2_ analog as reference (Li et al., 2005), the impact of elevated CaM on Kv7.2 is equivalent to a 5-fold increase in apparent PI(4,5)P_2_ binding affinity between the two states.

Co-expression of PIP5K led to a decrease in Kv7.3 current-density, and a reduction in the amount of protein from both total lysates and plasma membrane-isolated fractions. These observations could be explained by increased endocytosis and protein degradation. In our view, the role of PI(4,5)P_2_ on endocytosis (Balla, 2013; Posor et al., 2014) is the simplest explanation for the drop in Kv7.3 density by PIP5K. Current density depends on the probability of the channel being open (P_o_), the number of channels and the single channel conductance. Taking single channel conductance as constant (Li et al., 2005), the number of functional channels can be readily estimated when P_o_ approaches unity, such as it is the case for Kv7.3^T^ (Zaika et al., 2008). Thus, an 84% reduction in the number of channels can be inferred as a consequence of PIP5K expression. Kv7.2/3 P_o_ becomes closer to unity and that of Kv7.2 approaches 0.74 when tonic PI(4,5)P_2_ levels are elevated by PIP5K (Li et al., 2005). The drop in the number of Kv7.2/3 channels might have been compensated by the increase in P_o_, since the current density was not altered, in agreement with previous studies using another variant of HEK293 cells (Falkenburger et al., 2010). In contrast, Kv7.2 current density almost doubled with PIP5K, suggesting that the increase in P_o_ might have overshadowed the decrease in the number of channels. The tendency for diminution of Kv7.2 currents with PIP5K under decreased CaM levels was unexpected, because under resting CaM conditions PIP5K almost doubled the current. In other words, under resting CaM conditions the increase in P_o_ which follows the elevation of PI(4,5)P_2_ concentration outweighs the macroscopic current decrease expected from endocytosis, but this is not the case when CaM is scarce.

**Figure 10:**
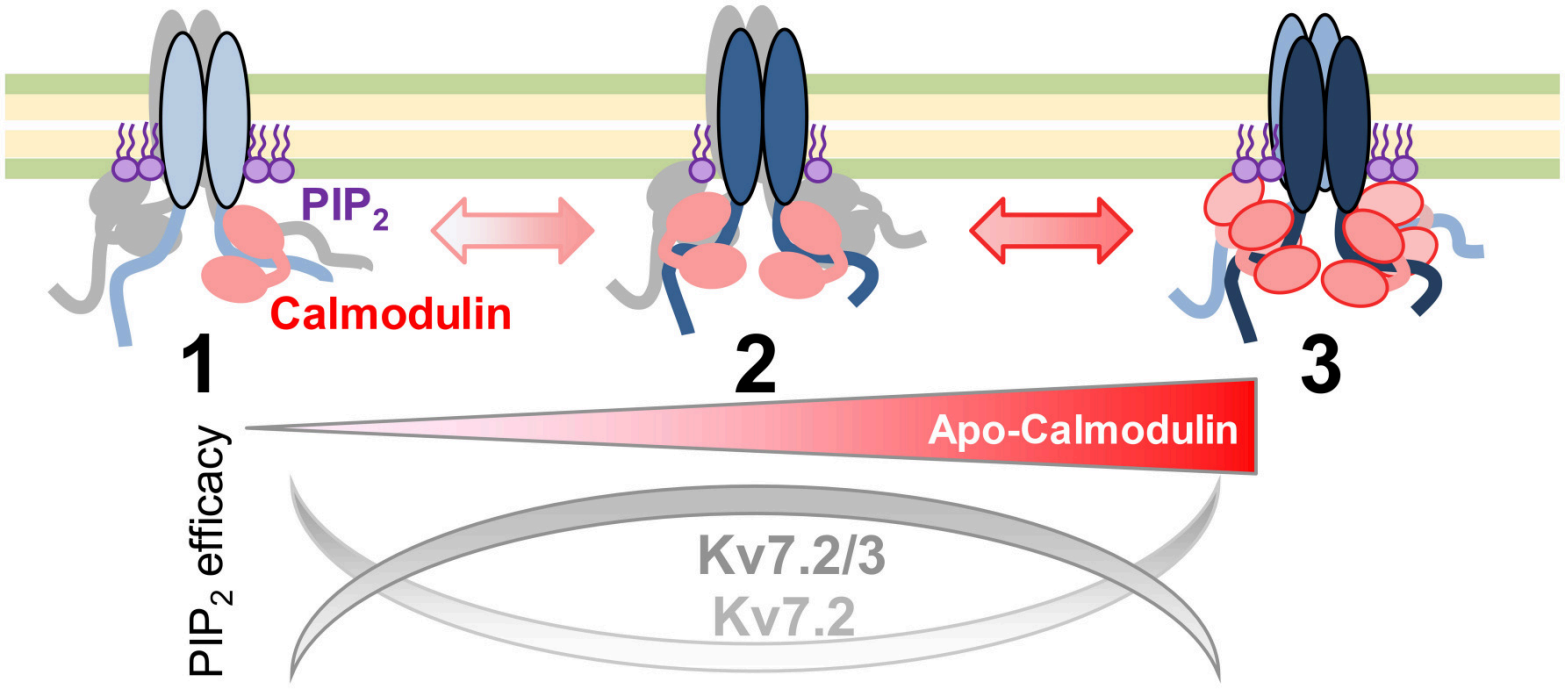
**Model of apocalmodulin regulation of Kv7 PI(4,5)P_**2**_ efficacy**. Illustration of the proposed equilibrium between three functional Kv7 channels pools. The PI(4,5)P_2_ efficacy depends upon the abundance of apoCaM and subunit composition. Pool 1 and pool 3 represent the currents observed after expression of a CaM sponge or overexpression of CaM_1234_, respectively.

The sensitivity to the actions of DrVSP and PIP5K suggests the existence of three functional pools of Kv7.2 channels in equilibrium as a function of CaM abundance (Figure 10). The main group observed when cells are not subjected to any change in CaM abundance appears to be the most dependent on PI(4,5)P_2_. When CaM becomes scarce, the pool of channels presents similar dependency on PI(4,5)P_2_ as when CaM is abundant. The conductance-voltage relationship differentiates these two pools. Free apoCaM levels are dynamically controlled in the nervous system by CaM-binding proteins, such as GAP43 (neuromodulin) and neurogranin (Xia and Storm, 2005), the latter being concentrated in dendritic spines. Upon neurogranin phosphorylation, apoCaM is released, having a major impact on long term potentiation of synaptic transmission (Neuner-Jehle et al., 1996; Ramakers et al., 1999; Huang et al., 2004; Zhabotinsky et al., 2006; Zhong et al., 2009). When the levels of apoCaM are elevated or CaM becomes phosphorylated, our model proposed that the equilibrium moves toward the pool on the right, with higher resistance to PI(4,5)P_2_ depletion. Under limited CaM conditions, there is a shift toward a different population, which also displays enhanced resistance to PI(4,5)P_2_ depletion. By moving between pools, the dependency for PI(4,5)P_2_ and the response to intracellular Ca^2+^ will be coordinated.

Thus, physiological or pathological oscillations of CaM abundance at diverse neuronal compartments will differentially affect the M-current depending on its molecular composition. This differential subunit-specific apoCaM/PI(4,5)P_2_ interplay may have profound physiological consequences considering the existence of Kv7.2 homomeric channels in certain neuronal domains (Kuba et al., 2015). In fact, homomeric Kv7.2 channels may contribute to M-channel diversity at some neuronal sites (Schwarz et al., 2006), particularly at early developmental stages (Devaux et al., 2004); neuropil subpopulations that display Kv7.2 but not Kv7.3 immunostaining are present in human brain sections (Cooper et al., 2000), and channels with high sensitivity to TEA and to Kv7.2 selective antibodies -probably Kv7.2 homomers- play a prominent role in hippocampal neurotransmitter release (Martire et al., 2004). This hypothetical mechanism may have substantial clinical impact, as Kv7 potassium channels strongly contribute to excitability in brain, heart, skeletal muscle and inner ear, and related diseases encompass epilepsy, autism, schizophrenia, cardiac arrhythmias, hearing loss, and sudden death (Jentsch, 2000; Soldovieri et al., 2011; Dvir et al., 2014; Maljevic and Lerche, 2014; Ambrosino et al., 2015). CaM abundance is decreased in depression disorders (Kang et al., 2012), and mutations in the CaM binding site of Kv7.2 are associated with distinct epilepsy phenotypes (Borgatti et al., 2004; Weckhuysen et al., 2012; Soldovieri et al., 2014). Changes in global or local CaM availability is expected to affect the response to agonist-mediated regulation. For instance, in sympathetic neurons, bradykinin receptor activation causes PI(4,5)P_2_ depletion, and a concomitant reduction of the M-current. Remarkably, this inhibition is prevented with both wt CaM and CaM_1234_ (Gamper and Shapiro, 2003; Zaika et al., 2007), an effect that could be explained by an increased resistance to PI(4,5)P_2_ depletion. It will be important to explore in detail the consequences of the mechanism unveiled here on neuronal excitability in normal and pathological conditions.

## Author contributions

CG: execution and interpretation; MS.: interpretation, novel reagents; CM: execution; PAm: execution; MT: conception, design, interpretation; PAr: interpretation; AV: conception, design, interpretation, writing the article. All authors approved the final version of the manuscript.

### Conflict of interest statement

The authors declare that the research was conducted in the absence of any commercial or financial relationships that could be construed as a potential conflict of interest.

## References

[B1] Alaimo A. Alberdi A. Gomis-Perez C. Fernández-Orth J. Gómez-Posada J. C. Areso P.. (2013). Cooperativity between calmodulin-binding sites in Kv7.2 channels. J. Cell Sci. 126, 244–253. 10.1242/jcs.114082 23203804

[B2] Alberdi A. Gomis-Perez C. Bernardo-Seisdedos G. Alaimo A. Malo C. Aldaregia J.. (2015). Uncoupling PIP2-calmodulin regulation of Kv7.2 channels by an assembly de-stabilizing epileptogenic mutation. J. Cell Sci. 128, 4014–4023. 10.1242/jcs.176420 26359296

[B3] Alexander K. A. Wakim B. T. Doyle G. S. Walsh K. A. Storm D. R. (1988). Identification and characterization of the calmodulin-binding domain of neuromodulin, a neurospecific calmodulin-binding protein. J. Biol. Chem. 263, 7544–7549. 2967288

[B4] Ambrosino P. Alaimo A. Bartollino S. Manocchio L. De Maria M. Mosca I.. (2015). Epilepsy-causing mutations in Kv7.2 C-terminus affect binding and functional modulation by calmodulin. Biochim. Biophys. Acta 1852, 1856–1866. 10.1016/j.bbadis.2015.06.012 26073431

[B5] Balla T. (2013). Phosphoinositides: tiny lipids with giant impact on cell regulation. Physiol Rev. 93, 1019–1137. 10.1152/physrev.00028.2012 23899561 PMC3962547

[B6] Black D. J. Tran Q. K. Persechini A. (2004). Monitoring the total available calmodulin concentration in intact cells over the physiological range in free Ca^2+^. Cell Calcium 35, 415–425. 10.1016/j.ceca.2003.10.005 15003851

[B7] Borgatti R. Zucca C. Cavallini A. Ferrario M. Panzeri C. Castaldo P.. (2004). A novel mutation in KCNQ2 associated with BFNC, drug resistant epilepsy, and mental retardation. Neurology 63, 57–65. 10.1212/01.WNL.0000132979.08394.6D 15249611

[B8] Bossuyt J. Bers D. M. (2013). Visualizing CaMKII and CaM activity: a paradigm of compartmentalized signaling. J. Mol. Med. 91, 907–916. 10.1007/s00109-013-1060-y 23775230 PMC3962229

[B9] Cooper E. C. Aldape K. D. Abosch A. Barbaro N. M. Berger M. S. Peacock W. S.. (2000). Colocalization and coassembly of two human brain M-type potassium channel subunits that are mutated in epilepsy. Proc. Natl. Acad. Sci. U.S.A. 97, 4914–4919. 10.1073/pnas.090092797 10781098 PMC18332

[B10] Devaux J. J. Kleopa K. A. Cooper E. C. Scherer S. S. (2004). KCNQ2 is a nodal K^+^ channel. J.Neurosci. 24, 1236–1244. 10.1523/jneurosci.4512-03.2004 14762142 PMC6793582

[B11] Dvir M. Peretz A. Haitin Y. Attali B. (2014). Recent molecular insights from mutated IKS channels in cardiac arrhythmia. Curr. Opin. Pharmacol. 15, 74–82. 10.1016/j.coph.2013.12.004 24721657

[B12] Etxeberria A. Santana-Castro I. Regalado M. P. Aivar P. Villarroel A. (2004). Three mechanisms underlie KCNQ2/3 heteromeric potassium M-channel potentiation. J. Neurosci. 24, 9146–9152. 10.1523/jneurosci.3194-04.2004 15483133 PMC6730048

[B13] Falkenburger B. H. Jensen J. B. Hille B. (2010). Kinetics of PIP2 metabolism and KCNQ2/3 channel regulation studied with a voltage-sensitive phosphatase in living cells. J. Gen. Physiol. 135, 99–114. 10.1085/jgp.200910345 20100891 PMC2812502

[B14] Gamper N. Li Y. Shapiro M. S. (2005). Structural requirements for differential sensitivity of KCNQ K^+^ channels to modulation by Ca^2+^/calmodulin. Mol. Biol. Cell 16, 3538–3551. 10.1091/mbc.e04-09-0849 15901836 PMC1182296

[B15] Gamper N. Shapiro M. S. (2003). Calmodulin mediates Ca^2+^-dependent modulation of M-type K^+^ channels. J. Gen. Physiol. 122, 17–31. 10.1085/jgp.200208783 12810850 PMC2234471

[B16] Gómez-Posada J. C. Aivar P. Alberdi A. Alaimo A. Etxeberría A. Fernández-Orth J.. (2011). Kv7 channels can function without constitutive calmodulin tethering. PLoS ONE 6:e25508. 10.1371/journal.pone.0025508 21980481 PMC3182250

[B17] Gómez-Posada J. C. Etxeberría A. Roura-Ferrer M. Areso P. Masin M. Murrell-Lagnado R. D.. (2010). A pore residue of the KCNQ3 potassium M-channel subunit controls surface expression. J. Neurosci. 30, 9316–9323. 10.1523/jneurosci.0851-10.2010 20610766 PMC6632484

[B18] Greene D. L. Hoshi N. (2017). Modulation of Kv7 channels and excitability in the brain. Cell. Mol. Life Sci. 74, 495–508. 10.1007/s00018-016-2359-y 27645822 PMC5243414

[B19] Huang K. P. Huang F. L. Jäger T. Li J. Reymann K. G. Balschun D. (2004). Neurogranin/RC3 enhances long-term potentiation and learning by promoting calcium-mediated signaling. J. Neurosci. 24, 10660–10669. 10.1523/jneurosci.2213-04.2004 15564582 PMC6730132

[B20] Jentsch T. J. (2000). Neuronal KCNQ potassium channels: physiology and role in disease. Nat. Rev. Neurosci. 1, 21–30. 10.1038/35036198 11252765

[B21] Jurado L. A. Chockalingam P. S. Jarrett H. W. (1999). Apocalmodulin. Physiol. Rev. 79, 661–682. 10390515 10.1152/physrev.1999.79.3.661

[B22] Kang H. J. Voleti B. Hajszan T. Rajkowska G. Stockmeier C. A. Licznerski P.. (2012). Decreased expression of synapse-related genes and loss of synapses in major depressive disorder. Nat. Med. 18, 1413–1417. 10.1038/nm.2886 22885997 PMC3491115

[B23] Kang S. Xu M. Cooper E. C. Hoshi N. (2014). Channel anchored protein kinase CK2 and protein phosphatase 1 reciprocally regulate KCNQ2-containing M-channels via phosphorylation of calmodulin. J. Biol. Chem. 289, 11536–11544. 10.1074/jbc.m113.528497 24627475 PMC4036288

[B24] Kosenko A. Kang S. Smith I. M. Greene D. L. Langeberg L. K. Scott J. D.. (2012). Coordinated signal integration at the M-type potassium channel upon muscarinic stimulation. EMBO J. 31, 3147–3156. 10.1038/emboj.2012.156 22643219 PMC3400014

[B25] Kuba H. Yamada R. Ishiguro G. Adachi R. (2015). Redistribution of Kv1 and Kv7 enhances neuronal excitability during structural axon initial segment plasticity. Nat. Commun. 6:8815. 10.1038/ncomms9815 26581625 PMC4673506

[B26] Li Y. Gamper N. Hilgemann D. W. Shapiro M. S. (2005). Regulation of Kv7 (KCNQ) K^+^ channel open probability by phosphatidylinositol 4,5-bisphosphate. J. Neurosci. 25, 9825–9835. 10.1523/jneurosci.2597-05.2005 16251430 PMC6725574

[B27] Liu X. Yang P. S. Yang W. Yue D. T. (2010). Enzyme-inhibitor-like tuning of Ca^2+^ channel connectivity with calmodulin. Nature 463, 968–972. 10.1038/nature09034 20139964 PMC3553577

[B28] Maljevic S. Lerche H. (2014). Potassium channel genes and benign familial neonatal epilepsy. Prog. Brain Res. 213, 17–53. 10.1016/b978-0-444-63326-2.00002-8 25194482

[B29] Martire M. Castaldo P. D'Amico M. Preziosi P. Annunziato L. Taglialatela M. M. (2004). channels containing KCNQ2 subunits modulate norepinephrine, aspartate, and GABA release from hippocampal nerve terminals. J. Neurosci. 24, 592–597. 10.1523/jneurosci.3143-03.2004 14736843 PMC6729253

[B30] Neuner-Jehle M. Denizot J. P. Mallet J. (1996). Neurogranin is locally concentrated in rat cortical and hippocampal neurons. Brain Res. 733, 149–154. 10.1016/0006-8993(96)00786-X 8891262

[B31] Posor Y. Eichhorn-Grunig M. Haucke V. (2014). Phosphoinositides in endocytosis. Biochim. Biophys. Acta 1851, 794–804. 10.1016/j.bbalip.2014.09.014 25264171

[B32] Putkey J. A. Sweeney H. L. Campbell S. T. (1989). Site-directed mutation of the trigger calcium-binding sites in cardiac troponin C. J. Biol. Chem. 264, 12370–12378. 2745448

[B33] Ramakers G. M. Gerendasy D. D. de Graan P. N. (1999). Substrate phosphorylation in the protein kinase Cgamma knockout mouse. J. Biol. Chem. 274, 1873–1874. 10.1074/jbc.274.4.1873 9890937

[B34] Sanabria H. Digman M. A. Gratton E. Waxham M. N. (2008). Spatial diffusivity and availability of intracellular calmodulin. Biophys. J. 95, 6002–6015. 10.1529/biophysj.108.138974 18820232 PMC2599858

[B35] Schwarz J. R. Glassmeier G. Cooper E. C. Kao T. C. Nodera H. Tabuena D.. (2006). KCNQ channels mediate I-Ks, a slow K^+^ current regulating excitability in the rat node of Ranvier. J. Physiol. 573, 17–34. 10.1113/jphysiol.2006.106815 16527853 PMC1779690

[B36] Shahidullah M. Santarelli L. C. Wen H. Levitan I. B. (2005). Expression of a calmodulin-binding KCNQ2 potassium channel fragment modulates neuronal M-current and membrane excitability. Proc. Natl. Acad. Sci. U.S.A. 102, 16454–16459. 10.1073/pnas.0503966102 16263935 PMC1283421

[B37] Sherman A. J. Shrier A. Cooper E. (1999). Series resistance compensation for whole-cell patch-clamp studies using a membrane state estimator. Biophys. J. 77, 2590–2601. 10.1016/s0006-3495(99)77093-1 10545359 PMC1300533

[B38] Soldovieri M. V. Ambrosino P. Mosca I. De Maria M. Moretto E. Miceli F.. (2016). Early-onset epileptic encephalopathy caused by a reduced sensitivity of Kv7.2 potassium channels to phosphatidylinositol 4,5-bisphosphate. Sci. Rep. 6:38167. 10.1038/srep38167 27905566 PMC5131271

[B39] Soldovieri M. V. Boutry-Kryza N. Milh M. Doummar D. Heron B. Bourel E.. (2014). Novel KCNQ2 and KCNQ3 mutations in a large cohort of families with benign neonatal epilepsy: first evidence for an altered channel regulation by syntaxin-1A. Hum. Mutat. 35, 356–367. 10.1002/humu.22500 24375629

[B40] Soldovieri M. V. Castaldo P. Iodice L. Miceli F. Barrese V. Bellini G.. (2006). Decreased subunit stability as a novel mechanism for potassium current impairment by a KCNQ2 C terminus mutation causing benign familial neonatal convulsions. J. Biol. Chem. 281, 418–428. 10.1074/jbc.m510980200 16260777

[B41] Soldovieri M. V. Miceli F. Taglialatela M. (2011). Driving with no brakes: molecular pathophysiology of Kv7 potassium channels. Physiology (Bethesda.) 26, 365–376. 10.1152/physiol.00009.2011 22013194

[B42] Suh B. C. Hille B. (2008). PIP2 is a necessary cofactor for ion channel function: how and why? Annu. Rev. Biophys. 37, 175–195. 10.1146/annurev.biophys.37.032807.125859 18573078 PMC2692585

[B43] Villarroel A. Taglialatela M. Bernardo-Seisdedos G. Alaimo A. Agirre J. Alberdi A.. (2014). The ever changing moods of calmodulin: how structural plasticity entails transductional adaptability. J. Mol. Biol. 426, 2717–2735. 10.1016/j.jmb.2014.05.016 24857860

[B44] Weckhuysen S. Mandelstam S. Suls A. Audenaert D. Deconinck T. Claes L. R.. (2012). KCNQ2 encephalopathy: emerging phenotype of a neonatal epileptic encephalopathy. Ann.Neurol. 71, 15–25. 10.1002/ana.22644 22275249

[B45] Winks J. S. Hughes S. Filippov A. K. Tatulian L. Abogadie F. C. Brown D. A.. (2005). Relationship between membrane phosphatidylinositol-4,5-bisphosphate and receptor-mediated inhibition of native neuronal M channels. J. Neurosci. 25, 3400–3413. 10.1523/jneurosci.3231-04.2005 15800195 PMC6724893

[B46] Xia Z. G. Storm D. R. (2005). The role of calmodulin as a signal integrator for synaptic plasticity. Nat. Rev. Neurosci. 6, 267–276. 10.1038/nrn1647 15803158

[B47] Xu T. Nie L. Zhang Y. Mo J. Feng W. Wei D.. (2007). Roles of alternative splicing in the functional properties of inner ear-specific KCNQ4 channels. J. Biol. Chem. 282, 23899–23909. 10.1074/jbc.m702108200 17561493

[B48] Zaika O. Hernandez C. C. Bal M. Tolstykh G. P. Shapiro M. S. (2008). Determinants within the turret and pore-loop domains of KCNQ3 K^+^ channels governing functional activity. Biophys. J. 95, 5121–5137. 10.1529/biophysj.108.137604 18790849 PMC2586577

[B49] Zaika O. Tolstykh G. P. Jaffe D. B. Shapiro M. S. (2007). Inositol triphosphate-mediated Ca^2+^ signals direct purinergic P2Y receptor regulation of neuronal ion channels. J. Neurosci. 27, 8914–8926. 10.1523/jneurosci.1739-07.2007 17699673 PMC6672180

[B50] Zhabotinsky A. M. Camp R. N. Epstein I. R. Lisman J. E. (2006). Role of the neurogranin concentrated in spines in the induction of long-term potentiation. J. Neurosci. 26, 7337–7347. 10.1523/jneurosci.0729-06.2006 16837580 PMC6674191

[B51] Zhang M. Tanaka T. Ikura M. (1995). Calcium-induced conformational transition revealed by the solution structure of apo calmodulin. Nat. Struct. Biol. 2, 758–767. 10.1038/nsb0995-758 7552747

[B52] Zhong L. Cherry T. Bies C. E. Florence M. A. Gerges N. Z. (2009). Neurogranin enhances synaptic strength through its interaction with calmodulin. EMBO J. 28, 3027–3039. 10.1038/emboj.2009.236 19713936 PMC2736013

[B53] Zhou X. Zhuang F. Li H. Zheng K. Hong Z. Feng W.. (2016). Calmodulin regulates KCNQ2 function in epilepsy. Am. J. Transl. Res. 8, 5610–5618. 28078031 PMC5209511

